# Anthocyanin Encapsulation as a Potential Approach for Improving the Quality of Aronia Powder

**DOI:** 10.3390/molecules31091523

**Published:** 2026-05-04

**Authors:** Senka Vidović, Milica Ramić Vasiljević, Rita Ambrus, Nataša Nastić, Nada Ćujić Nikolić, Teodora Janković, Aleksandra Gavarić

**Affiliations:** 1 Faculty of Technology, University of Novi Sad, Bulevar cara Lazara 1, 21000 Novi Sad, Serbia; senka.vidovic@uns.ac.rs (S.V.); natasa.nastic@uns.ac.rs (N.N.); 2 Fructus doo, Novosadski put 15/2, 21400 Bačka Palanka, Serbia; milica.vasiljevic@fructus.rs; 3 Department of Pharmaceutical Technology, University of Szeged, Eotvos 6, 6720 Szeged, Hungary; ambrus.rita@szte.hu; 4 Department for Pharmaceutical Research and Development, Institute for Medicinal Plant Research Dr. Josif Pančić, 11000 Belgrade, Serbia; ncujic@mocbilja.rs (N.Ć.N.); tjankovic@mocbilja.rs (T.J.)

**Keywords:** aronia powder, valorization, ultrasound extraction, spray drying, anthocyanins, flavonoids

## Abstract

Aronia fruit dust, generated in the industrial environment during processing, is considered a by-product discharged as waste, but it still contains high amounts of bioactive compounds such as polyphenolics, particularly anthocyanins. For the efficient isolation of anthocyanins and other flavonoids from this type of material, ultrasound-assisted extraction, at previously established optimal conditions, with a temperature of 70 °C, extraction time of 80 min, and ultrasonic power of 206 W, was applied. The extraction solvent was acidified with organic acids (citric, malic, and ascorbic acids) at low concentrations (4% and 8%) prior to spray drying to investigate the effects of the liquid feed pH value on the quality of the obtained aronia powders. Three anthocyanins—cyanidin-3-O-galactoside, cyanidin-3-O-arabinoside, and cyanidin-3-O-glucoside—along with the flavonoids rutin, hyperoside, and isoquercitrin were identified. The use of malic and citric acids in combination with maltodextrin produced aronia powders with a higher bulk density, smaller particle size, and more uniform particle size distribution compared to formulations containing ascorbic acid.

## 1. Introduction

In 2025, the global fruit powder market is estimated at approximately USD 19.1 billion, and it is projected to exceed USD 28 billion by 2030, reflecting a compound annual growth rate of around 7–8%. This growth is driven by the increasing consumer demand for natural, clean-label, and functional food ingredients, along with the expanding use of fruit powders in the food and beverage industry and the rising popularity of plant-based and health-oriented diets [1]. The production of high-quality fruit powders remains a challenge in spray drying, particularly for aronia-based feeds, which are rich in low-molecular-weight sugars and organic acids, resulting in low glass transition temperatures and pronounced stickiness during processing [2,3,4]. Additionally, the presence of sensitive valuable bioactive compounds, such as anthocyanins, and the tendency of dried particles to agglomerate further complicate the drying process. These challenges can be overcome through the careful optimization of process parameters and the appropriate selection of carrier agents and additional excipients. Such measures can contribute to stability in terms of lower agglomeration and improved physicochemical and morphological properties of the powders, which leads to enhanced applicability. However, the encapsulation efficiency and the resulting physicochemical and morphological properties of powders depend on the compositions of the liquid extracts to be encapsulated and the specific spray-drying parameters applied, such as the inlet/outlet temperature and flow rate, which are crucial. Furthermore, the scientific literature has documented that the inclusion of additional excipients alongside the selected carrier can, in certain cases, improve the overall quality of the final powder. For example, Ebrahimi et al. [5] reported the production of highly porous lactose with a pronounced degree of crystallinity, achieved through a process in which whey protein isolate was employed as the carrier and citric acid was added as an additional excipient used for the improvement of the powder’s properties. In another study by Shetty et al. [6], the medicine ciprofloxacin was co-spray-dried with disaccharides and L-leucine as additional excipients at a mass ratio of 1:1. They demonstrated an increase in the fine particle fraction compared with spray-dried ciprofloxacin alone. In another recent study by the same authors [7], the spray drying of the drug with the addition of lipid and phospholipid excipients was investigated, showing that lipids produced crystalline drug particles, whereas phospholipids produced partially amorphous drug particles. All of the co-spray-dried particles showed enhanced morphological characteristics in comparison to spray-dried drug particles without excipients.

Modern industry is continuously exploring novel resources to support the production of a wide range of products. This trend is evident in the powder production sector too. The use of alternative sources of nutrients and bioactive compounds, such as fruit waste or by-products, can contribute to the more efficient utilization of limited natural resources while promoting more sustainable production practices [8,9,10]. One such alternative source that can be used for the production of aronia (*Aronia melanocarpa* L.) spray-dried powder is aronia fruit dust (AFD), a by-product generated by the fruit filter tea industry. This by-product represents material of a lower particle size than the pores on the packaging material—the filter tea bag. As such, it cannot be used in further production and is eliminated from further processing.

Aronia is recognized as a superfruit due to its composition, being rich in polyphenols such as proanthocyanidins, anthocyanins, flavonols, and chlorogenic acids [11]. Aronia contains two main anthocyanins, cyanidin-3-galactoside and cyanidin-3-arabinoside, as identified in the studies by Horszwald et al. [12] and Samoticha et al. [13]. A recent dose–response meta-analysis revealed that taking purified anthocyanins at doses of 320 mg or more per day can lower serum levels of inflammation markers—specifically, C-reactive protein (CRP), interleukin-6 (IL-6), and tumor necrosis factor-alpha (TNF-α) [14]. Similarly, another study indicated that regularly consuming anthocyanin-rich foods can enhance vascular function, improve lipid profiles, and provide antioxidant and anti-inflammatory benefits [15]. Thus, from an application standpoint, it is necessary to preserve these compounds during any form of aronia processing, particularly if the product is required to retain the functional properties associated with the native material—aronia.

Therefore, the main objective of this study was to systematically evaluate the influence of the liquid feed composition on the properties of powders produced from aronia fruit dust (AFD) via ultrasound-assisted extraction followed by spray drying. Specifically, the effects of maltodextrin and selected organic acids (ascorbic, citric, and malic acid) as formulation additives were investigated. Powder characteristics were assessed in terms of the total phenolic content, anthocyanin concentration, and key physicochemical and morphological properties. Prior to this study, aronia fruit dust had not been systematically investigated as a feed material for spray drying in combination with multiple organic acids. While previous research has explored the stabilization of anthocyanins during processing, there is a lack of studies addressing the simultaneous use of different organic acids as protective agents during spray drying, particularly for complex, sugar-rich matrices such as aronia-based systems.

The novelty of this work lies in the application of three organic acids in combination with maltodextrin to enhance the stability of anthocyanins during the spray drying of aronia fruit dust. This approach provides new insights into formulation strategies aimed at improving the retention of sensitive bioactive compounds under high-temperature drying conditions, thereby contributing to the development of more stable, functional powder ingredients for food and related applications.

## 2. Results and Discussion

### 2.1. Process Efficiency and Sensory Properties of AFD Powders

The initial step in the production of aronia spray dry powders involves the preparation of the liquid feed, where the main compound is either cold-pressed aronia juice or aronia liquid extract. In the present study, the aronia ethanolic liquid extract was obtained from AFD using UAE. Successful applications of UAE for anthocyanins (the primary bioactive compounds in all aronia-based materials) have been reported in the literature, highlighting the advantages of UAE for the extraction of anthocyanins from elderberry, blackcurrant, blackberry, and cranberry [16,17,18,19]. In our previous studies, the efficiency of UAE applied for the isolation of phenolics and bioactive compounds from AFD was also confirmed [20,21]. Furthermore, in addition to the application of UAE, these studies also focused on the evaluation of the spray drying of aronia-based liquid feeds using laboratory-scale equipment. In these studies, the effects of maltodextrin of different dextrose equivalents (MD DE 19.7; MD DE 13.1; MD DE 5.9), applied at specific mass fractions (20, 40, 60%), on the quality of the obtained powders were investigated. The results indicated that MD DE 19.7 was the most efficient, yielding the powder with the highest total phenolic content and total monomeric anthocyanin content, along with favorable stability and physical properties. Based on these findings, MD DE 19.7 was selected as the primary carrier for the present study, which was conducted at a larger scale—namely using semi-industrial-scale equipment.

The present study also investigated the addition of organic acids—citric, malic, and ascorbic acids—to the liquid feed and their impacts on the physicochemical and morphological properties of the produced powders. The working hypothesis was that the acidification of the liquid feed at low concentrations (4% and 8%) prior to spray drying may contribute to (a) the potential stabilization of the liquid feed and powders—for example, through reduced enzymatic browning and polyphenol oxidase activity; (b) possible improvements in powder stability, including bioactive compound retention and reduced particle agglomeration; (c) changes in crystallinity; and (d) modified dissolution behavior in water. Citric, malic, and ascorbic acids were selected due to their common use in the food and beverage industry. Citric acid, in particular, serves multiple functions, including acidity regulation, preservation, antioxidant properties, emulsification, flavor and aroma enhancement, buffering, and antibacterial activity [22]. Malic and ascorbic acids are used for similar purposes and additionally act as plasticizers in biodegradable food packaging films. All three acids are highly soluble in water and ethanol solutions [23,24].

Although the addition of acids is expected to enhance certain powder properties, it may also introduce challenges during the spray-drying process itself. This is primarily due to the low glass transition temperatures of these acids, which can reduce the overall efficiency of the spray-drying process; this depends on their concentrations in the liquid feed. Additionally, according to Barra et al. [25], the thermo-oxidative degradation that occurs during spray drying in the presence of oxygen can accelerate acid loss. In contrast to the low glass transition temperatures of the low-molecular-weight sugars expected to be present in high-concentration aronia-based liquid feeds and the low glass transition temperatures of the acids applied, maltodextrins are characterized by high glass transition temperatures. These values depend on the dextrose equivalent; as an example, for maltodextrin of DE 20, it is 141 °C, while, for maltodextrin of DE 5, it is 188 °C. Therefore, it can be assumed that an appropriate combination of maltodextrin and the selected acid, if optimized correctly, could enhance the powder particle properties and, at the same time, prevent the degradation of the targeted bioactive compounds as well as the applied acids, owing to maltodextrin’s protective effects and its high glass transition temperature.

The results indicated that the spray-drying process was not efficient at all when maltodextrin was applied at concentrations of 20% and 40%. The process efficiency is presented in Table 1.

Meanwhile, with maltodextrin increased to a concentration of 100%, the efficiency increased to 58.9%, which is generally considered acceptable for the spray-drying process. A further increase in the carrier concentration to 150% led to even higher process efficiency of 74.0%. In powder samples P2–P7, where maltodextrin was used in combination with organic compounds, the process efficiency ranged from 70.3% (P2) to a maximum of 78.5% (P5), which was obtained in the case of malic acid addition. Generally, higher efficiency was achieved when higher concentrations of acids were combined with maltodextrin.

As noted above, an inefficient drying process was observed when powders were produced using concentrations of 20% and 40%, characterized by the formation of a sticky layer on the inner walls of the drying chamber and the connecting tube between the chamber and cyclone. Aronia berries naturally contain higher concentrations of simple sugars, which are also present in commercially available berries such as strawberries, blueberries, blackberries, currants, and raspberries. Notably, aronia also contains substantial levels of sorbitol—a naturally occurring polyol—commonly used in the food industry for its sweetening, humectant, and texturizing properties [26]. Thus, the inefficiency of the process can be explained by the high concentrations of these low-molecular-weight sugars, including sorbitol.

Visual appearance is one of the key sensory characteristics, particularly when AFD powder is intended for use as a natural food colorant. All seven AFD powders—i.e., P1, obtained using MD, and P2–P7, obtained by combining MD and organic acids—exhibited a satisfactory visual appearance, with the intensity of the characteristic purple color remaining and being unaffected by the addition of various organic acids. According to the literature, the currently available enzymatic browning inhibitors in the food industry are ascorbic acids and their derivatives, organic acids, sulfur-containing amino acids, and their combinations [27]. Ascorbic acid, a potent water-soluble antioxidant, has been extensively employed in the food industry due to its efficiency in preventing the rancidity of unsaturated fats and in protecting nutrients from oxidative degradation [28]. Additionally, the advantage of the potential addition of organic acids to the liquid feed is that colloidal particles, such as pectin, proteins, and hemicelluloses, can bind to the precipitated ions formed by the organic acid, facilitating their removal during filtration and resulting in reduced turbidity—a process that is applicable in juice clarification [29]. Thus, the addition of organic acids to the AFD liquid feed can have several beneficial effects.

### 2.2. Physical Properties of AFD Powders

#### 2.2.1. Moisture Content, Hygroscopicity, and Bulk Density

The results regarding the powders’ physical properties, such are moisture content, hygroscopicity, and bulk density, are presented in Table 2. The moisture content of the spray-dried AFD powders ranged from 3.39 to 5.46%. Considering the moisture content as a quality parameter, moisture content of less than 5% is required to ensure the long-term stability, efficient packaging, and storage of the obtained powder [30]. Such values indicate a low risk of microbial spoilage and consequently an adequate shelf life. All powders, with the exception of P100 and P1—which were produced using only maltodextrin—exhibited moisture content below 5.0%. These results suggest that the combination of organic acids with maltodextrin had a beneficial effect in reducing the moisture content, thereby contributing to the production of powders with improved quality in terms of moisture content. The most positive effect was observed with the addition of citric acid at the concentration of 8%, which yielded the lowest moisture content, below 3.5%. It is clear that the combination of the selected acids with maltodextrin DE 19.7 and drying at 140 °C (selected as optimal in previous studies) resulted in lower-moisture powders. In a study by Nadali et al. [31], it was found that the inlet temperature and maltodextrin DE had significant individual and combined effects on the moisture content of barberry juice powder. The inlet temperature plays a major role in spray-dried powders’ moisture content. The enhanced heat transfer rate as a consequence of a higher inlet temperature provides a greater driving force, which can lead to the production of dried particles with lower moisture content [32,33]. Regarding the impact of the DE, Fazaeli et al. [34] reported that higher DEs (DE6  <  DE9  <  DE20) resulted in increased moisture content in spray-dried black mulberry powders. They also stated that maltodextrins with lower molecular weights contained shorter chains and increased hydrophilic groups. Thus, both these parameters impact powder quality. The fact that an increase in MD concentration is accompanied by an increase in solid matter in the feed solution and a decrease in total moisture to be evaporated may result in a decrease in the total moisture content of the final product [35]. This is also in accordance with previous results reported for spray-dried commercial aronia juice, as well as aronia juice and wine encapsulated with maltodextrin [36].

The results regarding powder hygroscopicity are presented in Table 2. According to the results, the samples produced with organic acid addition (P2–P7) did not exhibit any relevant differences in hygroscopicity. Water absorption with increased time (120 h) was noted in each sample, resulting in the maximum hygroscopicity of 16.60% detected in P7—the powder obtained with the addition of ascorbic acid at a concentration of 8%. This difference in water adsorption is related to the number of hydrophilic groups present in the structure of each agent. The phenomenon of water adsorption by carbohydrates is attributed to the links between the hydrogen present in water molecules and the hydroxyl groups available in the amorphous regions of the substrate, as well as on the surfaces of crystalline regions. Maltodextrin has a large number of links with hydrophilic groups and therefore can absorb moisture from the ambient air. In addition, the differences in the hygroscopicity of the samples can be attributed to the sizes of the particles produced with each of the agents, which will be further discussed in Section 2.2.3 regarding the particle size distribution. The larger the particle size, the smaller the exposed surface area and, consequently, the lower the water adsorption from the ambient air. Similar results were obtained by other authors [37] regarding the microencapsulation of betacyanins and anthocyanins using maltodextrins with different dextrose equivalents. These authors verified an increase in hygroscopicity with increasing DEs and attributed such an increase to the lower molecular weights of the maltodextrins with higher DEs, which have shorter chains and therefore more hydrophilic groups.

Bulk density is an important physical property with which to determine how a powder ingredient will blend with other, solid-form constituents, whereas the particle size of feed generally influences the mixing process. A higher bulk density is convenient for reducing shipping and packaging costs. According to Phisut et al. [38], once the droplet’s water content reaches a critical threshold, a dry crust develops on its surface, causing the drying rate to sharply decline as the drying front advances. At this stage, the rate of drying relies on the diffusion of water through this newly formed crust. The speed of a powder’s crust formation can affect the particle size and density, which are assumed to increase with an increment in feed concentration. However, an increase in the feed’s solid concentration may also cause a reduction in particle density, probably due to rapid crust formation, which prevents water from reaching the surface. Usually, increasing the feed concentration produces a decrease in bulk density due to the increase in particle size, although the opposite effect has also been observed [18]. In this study, the above-mentioned trend was observed: with an increase in the MD concentration from 100% to 150%, the bulk density decreased from 90.62 to 73.68 mg/mL, respectively. The bulk density is around 77 mg/mL when citric and malic acids are used at lower concentrations in combination with maltodextrin, but it decreases when citric and malic acids are added at an 8% concentration.

#### 2.2.2. Water Solubility Index and Water Absorption Index Results

The hydration properties of AFD powders can be described by the water solubility index (WSI) and the water absorption index (WAI). The WSI is a key indicator of how a product behaves in the aqueous phase and serves as a general criterion for assessing the reconstitution quality of powders. For consumers, the ability of powdered products to dissolve and be reconstituted quickly and completely in the aqueous phase is a primary quality indicator [39,40]. However, in this study, increasing the maltodextrin concentration from 100 to 150% caused a decrease in powder solubility of 10% (Table 3). This result is consistent with those obtained by Moreira et al. [41], who reported that the water solubility of acerola pomace extract powders was negatively affected by maltodextrin, although the solubility remained above 90% in powders across all treatments. According to our previous study, the WSI of aronia powders dried at 140 °C with the addition of MD DE 19.7 ranged from 56 to 65% [20,21], while, in the present study, the WSI was notably improved, probably due to the addition of a higher mass fraction of maltodextrin. The lower WAI suggests greater stability during storage, as it indicates that the powder is less likely to absorb moisture and degrade over time. It is influenced by the presence of hydrophilic groups, which bind to water molecules, and the gel-forming capacity of macromolecules [42].

Regarding the WAI, the values were quite similar, being independent of the organic acid type and concentration used. Usually, the WAI and WSI are negatively and positively correlated with the MD, respectively. However, according to our previous study, the WAIs of AFD powders with the addition of MD DE 19.7 at different ratios (20, 40, 60%) ranged from 0.0030 to 0.0035 g/g [20,21], while, in our present study, after the addition of the same MD to 150%, the WAI increased to 0.75–0.82 g/g. This could be explained by the nature and chemical structure of maltodextrin and the number of hydroxyl (-OH) groups, which have a strong affinity for water, allowing water to bind and eventually causing water to be retained in the powder itself. Organic acid addition had a negligible effect on both the WSI and WAI of the obtained AFD powders.

#### 2.2.3. Particle Size Distribution

The particle size of a spray-dried powder depends on the liquid feed, the type of encapsulating agent and other agents, the drying technique used, and specific process parameters. Typically, the particle size in spray-dried powders ranges from 10 to 100 µm. The particle size of a powder, measured as the average particle diameter, is a critical factor in determining the powder’s utility and potential application in food matrices. In food powder production, one of the primary quality indicators for both manufacturers and consumers is the rapid and complete reconstitution of the powdered ingredients. The functional and reconstitution properties of these powders are significantly influenced by the specific surface area (SSA) [43,44]. The preferred particle size generally depends on the type of application; in some cases, smaller particles are desirable, while, in others, larger particles are favored. In both cases, a uniform particle size distribution is advantageous.

The size distributions of AFD powders produced with different loading content were determined using microscopic measurements, and the results are summarized in Table 4. The standard percentiles D (0.1), D (0.5), and D (0.9), or the sizes of particles below which 10%, 50%, and 90% of the samples lay, were also considered for powder characterization. The microparticles in various AFD powders consisted predominantly of particles having diameters in the range of 6–9 μm. The results indicate that the combination of organic acids with maltodextrin influenced the particle sizes of the powders, with the most pronounced effects observed for formulations containing ascorbic acid at both tested concentrations. Specifically, powders P6 and P7 exhibited significantly larger particle sizes, as reflected by the increased D (0.5) and D (0.9) values. However, this observation is not attributed to ascorbic acid thermal degradation, as no direct evidence supports this mechanism. Instead, differences in feed properties, such as rheology, or variations in atomization behavior during spray drying may have contributed to the observed particle size distribution. The results showed that more than 90% of particles were smaller than 25 µm and 32 µm in the P6 and P7 powders, respectively. The difference between D (0.1) and D (0.9) reflects the uniformity of the particle size distribution. Based on the obtained results, the most uniform particle distribution was observed in powders produced using maltodextrin at the concentration of 150%. A similar degree of uniformity was achieved with the combination of maltodextrin and citric acid (P2, P3, P4, and P5). In contrast, a more significant gap—a wider size distribution and lower uniformity—was noted in the powders produced with ascorbic acid addition at both tested concentrations; this may have been induced by certain changes in the physical properties of ascorbic acid itself during spray drying, e.g., thermal degradation or instability. In contrast to ascorbic acid, citric and malic acids exhibited greater thermal stability, which contributed to the more uniform particle size distributions observed in powders P2–P5.

### 2.3. Interpretation of DSC Peaks in Terms of Crystallinity and Decomposition

**Figure 1:**
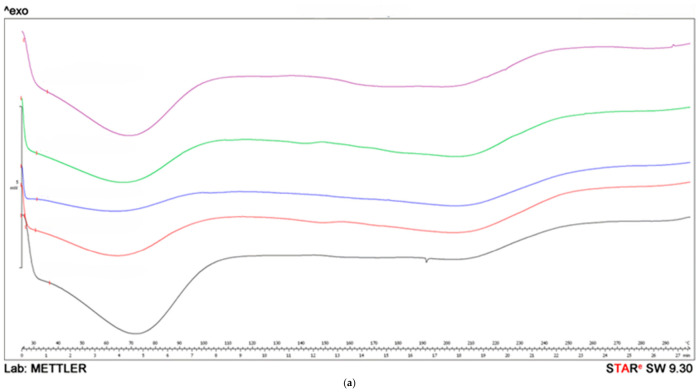

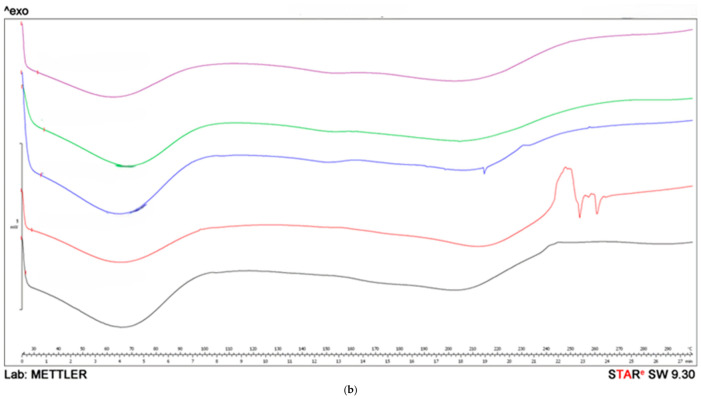

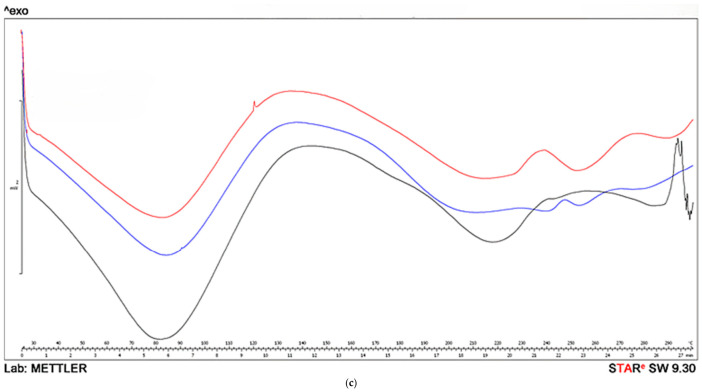
DSC thermograms of the AFD powders: (**a**) P020 (purple), P040 (green), P100 (blue), P1 (red), P2 (black); (**b**) P3 (purple), P4 (green), P5 (blue), P6 (red), P7 (black); and (**c**) organic acids, namely malic (red), citric (blue), and ascorbic (black).

Differential scanning calorimetry (DSC) is a helpful tool to describe the thermal transitions of AFD powders at a micro scale. This analysis offers insights into the thermodynamic and kinetic properties as influenced by the material temperature. Such insights can prove valuable in assessing the shelf lives of powdered products and understanding their rheological behavior [45,46,47]. According to the thermo-analytical measurements, the DSC thermograms (Figure 1a–c) indicate that the analyzed powders are predominantly amorphous or only weakly semi-crystalline, with the observed thermal events mainly reflecting physical transitions rather than chemical decomposition.

The broad endothermic region at low temperatures (≈40–110 °C) can be attributed to the loss of adsorbed and weakly bound water, with its greater intensity in some samples suggesting a higher amorphous fraction and a more heterogeneous water-binding environment. In the intermediate temperature range (≈120–180 °C), the absence of a sharp melting peak and the presence of a wide, smooth thermal event indicate structural relaxation and the reorganization of disordered domains, consistent with low crystallinity rather than the melting of a well-defined crystalline phase. Since there are no sharp diffraction peaks in the curves, it can be concluded that the crystalline structure of the active constituents and the matrix did not develop in all investigated powders. Figure 1b also shows the DSC curve for the AFD powder with 4% ascorbic acid (P6), with an exothermic peak at a higher temperature (∼250 °C), related to an oxidative decomposition process—most likely the thermal degradation of ascorbic acid. The absence of the corresponding exothermic peak at higher temperatures in sample P7 was not expected, particularly since a similar thermal event was observed for pure ascorbic acid; see Figure 1c. This suggests the possibility of interactions among the formulation compounds within P7, which may have enhanced the thermal stability of the ascorbic acid used; thus, deeper investigation regarding this observation is needed. Overall, the DSC profiles confirm the low structural order and good thermal stability of the samples, with crystallinity-related phenomena dominating the thermal behavior, rather than decomposition processes.

**Figure 2:**
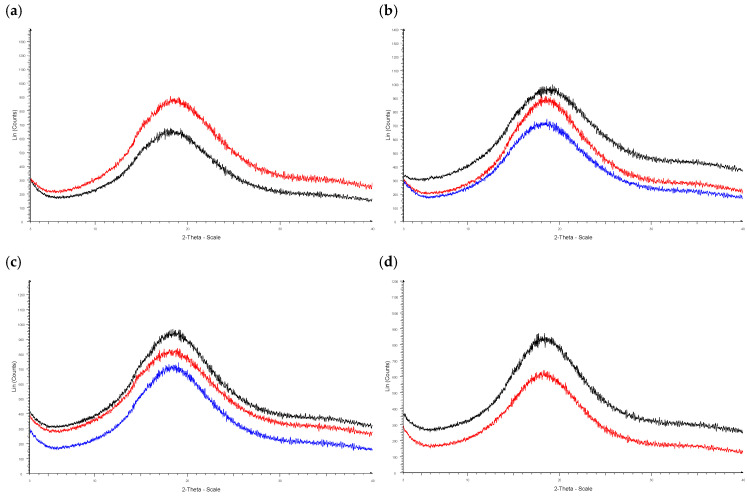
XRPD diffractograms of the AFD powders: (**a**) P020 (red), P040 (black); (**b**) P1 (blue), P100 (red), P2 (black); (**c**) P4 (blue), P3 (red), P5 (black); (**d**) P6 (red), P7 (black).

The XRPD analysis confirmed that the AFD powders were X-ray-amorphous, since there were no sharp diffraction peaks that indicated the crystallinity of the tested samples (Figure 2a–d) [46,47]. All powder patterns exhibited typical features of amorphous substances, with a broad, diffuse signal centered at about 18–19° at a 2θ scale, as seen in a previously published study where XRPD diffractograms were described for maltodextrin-based aronia microparticles [20,21]. Minor differences in the position, intensity, and shape of the diffuse signals can primarily be attributed to variations in sample morphology caused by differences in powder compressibility.

### 2.4. Anthocyanins and Flavonoids in AFD Liquid Feeds and Powders

Since anthocyanins are prone to degradation, recently, there have been numerous attempts to extract them from aronia using different green extraction technologies, such as using deep eutectic solvents and plasmolyzed yeast cells [48,49]. In our study, to further evaluate anthocyanin degradation during UAE, an additional set of experiments was performed. Ultrasound-assisted extraction at 70 °C was conducted at different extraction times (60, 80, 100, and 120 min). The highest levels of total phenolics, flavonoids, and anthocyanins were obtained at 100 min. To further confirm these findings, HPLC-DAD analysis was performed, which verified that the predominant anthocyanins (Cya-gal, Cya-glu, and Cya-ara), as well as chlorogenic acid and flavonoids (rutin, hyperoside, and isoquercitrin), were present at their highest levels at 100 min. In line with these results, Mahdavi et al. [50] reported that approximately 80–90% of the extracted anthocyanins are subsequently processed by spray drying for encapsulation and the production of natural food colorants.

In the present study, the chemical profiles of the AFD liquid feeds, obtained via UAE, and their corresponding spray-dried powders were analyzed via the HPLC-DAD method. The concentrations of anthocyanins detected in the AFD liquid feeds and the powders are presented in Table 5, while the concentrations of their phenolic compounds are shown in Table 6. Three anthocyanins, namely cyanidin-3-O-galactoside (Cya-Gal), cyanidin-3-O-arabinoside (Cya-Ara), and cyanidin-3-O-glucoside (Cya-Glu), along with flavonoids including quercetin-3-O-rutinoside (rutin), quercetin-3-O-galactoside (hyperoside), and quercetin-3-O-glucoside (isoquercitrin), as well as chlorogenic acid (CA), were determined before and after the spray-drying process to assess the influence of the inlet temperature (140 °C) and liquid feed formulation composition on the stability. Catalkaya et al., 2022 [51] also detected three main anthocyanins, cyanidin-3-galactoside, cyanidin-3-glucoside, and cyanidin-3-arabinoside, in all tested powders derived from black chokeberry pomace.

In all AFD liquid feed formulations, the most abundant anthocyanin was Cya-Gal, followed by Cya-Ara, present in around two-fold-lower concentrations in comparison to Cya-Gal. Numerous reports on the compositions of aronia anthocyanins have identified Cya-Gal as the most abundant compound across various types of aronia products, including the fruit, juice, and pomace. Cya-Gal mainly occurs in fruits, including red-skinned or red-fleshed apples, hawthorn, bilberries, cranberries, lingonberries, and aronia berries. This natural anthocyanin is reported as a major component of black, purple, and red aronia, being the highest in black aronia. It has many beneficial health effects, including a strong antioxidative capacity and the ability to decrease LDL cholesterol and triglyceride levels, and its oral administration could improve spatial memory in mice [52]. Additionally, Cya-Ara is consistently reported as the second-most-prevalent anthocyanin, which aligns with our findings. This observation does not necessarily reflect a true increase in phenolic content but may instead result from several factors. Acidification can enhance the extractability and solubility of phenolic compounds by promoting cell wall disruption or improving mass transfer. Additionally, lower-pH conditions may stabilize certain phenolics (particularly anthocyanins), reducing their degradation during processing and thus leading to higher measured concentrations. It is also possible that analytical factors, such as changes in matrix interactions or improved chromatographic responses under acidic conditions, contributed to the observed differences.

Scientific studies are showing that the addition of acids to liquid systems can improve their stability. As an example, according to Hubbermann et al. [53], the addition of citric acid can enhance anthocyanins’ stability by reducing the hydration rate due to its low dissociation constant. According to Zhao et al. [54], ascorbic acid has the potential to enhance the stability of anthocyanins if it is combined with rosmarinic acid and xanthan gum. In the present study, the highest concentration of the major anthocyanin, Cya-Gal (0.84 mg/mL), was detected in sample L7. However, this observation should be interpreted with caution, as it does not necessarily confirm the stabilization effect of ascorbic acid at the applied concentration (8%) in combination with maltodextrin (DE 19.7). The observed result may reflect formulation- or analytical-related factors, and therefore no definitive conclusion regarding anthocyanin stabilization can be drawn. In the liquid feed L1, which contained the same concentration of maltodextrin as L7, the corresponding concentration of Cya-Gal was significantly lower at 0.52 mg/mL. A similar observation was made in the case of the second-most-dominant anthocyanin, Cya-Ara. In contrast, ascorbic acid added at a lower concentration did not exhibit the same positive effect as at a higher concentration; in fact, here, a negative effect was observed, reducing the Cya-Gal concentration to around two-fold lower. The addition of malic and citric acids to the liquid feed containing a constant maltodextrin concentration (150%) did not produce a significant effect, as no significant change in Cya-Gal concentration was observed at both tested acid concentrations. Regarding phenolic compounds, the dominant one detected in all liquid feeds was chlorogenic acid, while the detected concentrations of others were quite low.

A common characteristic of all AFD powders is that the levels of anthocyanins and phenolic compounds are notably reduced after the encapsulation process, likely due to thermal degradation occurring at 140 °C. According to the results in Table 5 and Table 6, despite the fact that the anthocyanins were less degraded than the phenolic compounds, they were recovered in the powders at two- to three-fold-lower concentrations than in their liquid analogs. However, the greatest deterioration was seen for P7, probably due to the facilitated degradation of anthocyanins in the presence of oxygen within the air, which was used as a drying medium in the spray-drying process.

## 3. Materials and Methods

### 3.1. Chemicals

Folin–Ciocalteu reagent (Sigma-Aldrich, Steinheim, Germany), sodium chloride 99.5% (Lach-Ner, Neratovice, Czech Republic), and sodium carbonate (Centrohem, Stara Pazova, Serbia) were used, as well as gallic acid, methanol, formic acid, orthophosphoric acid, and potassium bromide, which were obtained from Sigma-Aldrich (Steinheim, Germany). Ethanol was of analytical grade; acetonitrile (Merck, Darmstadt, Germany) was of HPLC grade; and ultra-pure water was prepared using a Milli-Q purification system (Millipore, Guyancourt, France). The standards of cyanidin-3-O-galactoside, cyanidin-3-O-glucoside, cyanidin-3-O-arabinoside, rutin, hyperoside, isoquercitrin, and chlorogenic acid were purchased from Extrasynthese (Genay, France). Citric, malic, and ascorbic acids were of food-grade quality. Maltodextrin DE 19.7 was supplied by Brenntag (Mülheim, Germany).

### 3.2. Plant Material

In the filter tea industry, dried aronia (*Aronia melanocarpa* L.) cake—remaining after juice extraction in the beverage industry—is further processed into a dried fruit fraction, which is used as an ingredient in various herbal and fruit blends packaged in filter tea bags. Prior to blending with other components, aronia cake undergoes several processing steps, including milling, grinding, and fractionation. During these operations, around 30% of the input plant material is of a particle size that is lower than the pore size (<0.315 mm) of the filter tea bag and is designated as aronia fruit dust (AFD). Since this type of material cannot be utilized for the further production of the final product in the form of filter bags, it is discharged as waste. The AFD used in this study was donated by Fructus d.o.o. (Backa Palanka, Serbia), a producer of herbal and fruit filter teas. The moisture content of the aronia fruit dust was 8.7%.

### 3.3. Preparation of AFD Extract

Ultrasound-assisted extraction (UAE) was selected as the method used to obtain the liquid extract using AFD, with 50% ethanol as an extraction solvent and an S/L ratio of 1:5 (*w*/*v*). Extraction parameters were set according to the previously published study by Vidović et al. [20], in which the optimal conditions for UAE from AFD were determined as a temperature of 70 °C, extraction time of 80 min, and ultrasonic power of 206 W. Extraction was performed in a sonication water bath (EUP540A, EU Instruments, Paris, France) with a fixed frequency at 40 kHz. After the set extraction time, the obtained extract was immediately filtered under a vacuum, collected into a glass flask, and stored at 4 °C until further processing.

### 3.4. Preparation of Liquid Feeds

For the preparation of AFD liquid feeds, maltodextrin (MD) of dextrose equivalent (DE) 19.7 was added to the obtained liquid extract as a carrier. This mixture was stirred using a magnetic stirrer under a temperature of 40 °C. The first three samples—liquid feeds—were prepared with the addition of MD 19.7 DE at concentrations of 20, 40, and 100% (calculated based on total solids content). All other liquid feeds were prepared with the addition of 150% MD 19.7 DE, also calculated on the basis of the total solids content. Besides MD 19.7 DE, prior to spray drying, three organic acids, namely citric acid, malic acid, and ascorbic acid, were also added as excipients at concentrations of 4% or 8%, calculated based on the total solids content. After addition, the pH value of each AFD liquid feed was measured with a WTW inoLab pH 7110 pH meter. The specific designations of the liquid feeds and their powder analogs are presented in Table 7.

### 3.5. Degradation of Anthocyanins in Liquid Feed

An additional set of experiments was conducted to evaluate the degradation behavior of anthocyanins during UAE. Specifically, ultrasound-assisted extraction with 50% ethanol as an extraction solvent and an S/L ratio of 1:5 (*w*/*v*) at 70 °C was repeated at different extraction times (60, 80, 100, and 120 min). The total phenolic and total flavonoid content [55,56] and the total anthocyanin content [57] were determined in AFD extracts without maltodextrin or organic acid addition, while anthocyanin concentrations were quantified using HPLC-DAD, enabling accurate analysis directly in the liquid extracts. These results (Table 8, Table 9, Table 10, Table 11 and Table 12) provide a more relevant assessment of anthocyanin stability under actual extraction conditions, taking into account both the effects of ultrasound and interactions within the solvent system.

### 3.6. Spray Drying of Liquid Feeds

All AFD liquid feeds were dried at a constant inlet and outlet temperature of 140 °C and 80 ± 2 °C, respectively, using a pilot-scale spray dryer (APV Anhydro AS, Soeborg, Denmark). A peristaltic pump was employed to transport the prepared AFD liquid feed from the container into the drying chamber. Spray drying was performed in a co-current flow. Atomization was accomplished using an atomizer operating at speeds between 20,000 and 21,000 rpm. The rate of liquid feed during the drying process was 1.36 L/h. Powder particles were separated from the outlet air using a cyclone separator. Each batch of AFD powder was collected in a high-density polyethylene zip-lock plastic bag and stored in the dark at −20 °C until further analyses. The process efficiency (PE) was calculated according to the following equation:

PE (%) = (practical mass of collected powder/theoretical mass of total solids in the feed) × 100(1)

where the theoretical mass was determined based on the total solids content in the feed formulation prior to spray drying, and the practical mass represents the amount of powder recovered after the process.

Regarding the experimental design, each formulation was spray-dried in independent runs, rather than being only analytically replicated.

### 3.7. Analyses of Powders’ Physical Properties

#### 3.7.1. Moisture Content

The moisture content in the obtained powders was measured immediately after the spray-drying process. It was performed thermogravimetrically according to the standard procedure by drying a powder sample in an oven at 105 °C until a constant mass. All experiments were performed in three replicates.

#### 3.7.2. Hygroscopicity

The hygroscopicity of the obtained powders was determined according to the modified method of Cai and Corke [58] and Goula and Adamopulos [59]. Approximately 1 g of each powder, precisely weighted, was placed at room temperature in an air-tight container or desiccator filled with a NaCl saturated solution (70% RH). Hygroscopicity was monitored after 48 h and 120 h, and it was expressed as g of absorbed water per 100 g of powder (g/100 g). All experiments were performed in three replicates.

#### 3.7.3. Bulk Density

During bulk density analysis, a sufficient quantity of powder to complete the test should be passed through a sieve with an aperture greater than or equal to 1.0 mm in order to break up any agglomerates that may have formed during storage; this should be performed gently to avoid altering the nature of the material. Approximately 1 g (m) of the test sample, weighed with 0.1 percent accuracy, should then be gently introduced into a dry, graduated 25 mL cylinder (readable to 2 mL), without compaction. If necessary, the powder should be carefully leveled, again without compaction, and the unsettled apparent volume (V_0_) should be read to the nearest graduated unit. The bulk density, in milligrams per milliliter, is then calculated using the formula m/V_0_.

#### 3.7.4. Water Solubility Index and Water Absorption Index

The water solubility index (WSI) and water absorption index (WAI) of the powders were determined according to the experimental protocol described by Phoungchandang and Sertwasana [60]. Briefly, 1.25 g of powder and 15 mL of water were vigorously mixed in a 50 mL centrifuge tube and incubated in a water bath for 30 min at 30 °C. After incubation, the mixture was centrifugated (LC-320, Tehtnica, Zelezniki, Slovenia) at 3000× *g* for 15 min. The supernatant was decanted in a pre-weighed Petri dish, while the solid particles formed a pellet at the bottom of the tube. Both the supernatant and the pellet were dried overnight at 105 °C. The WSI was calculated as the percentage of solids in the dried supernatant relative to the total dry solids in the original 1.25 g of sample. The WAI was determined by dividing the mass of solid pellets by the mass of the original dry sample. All experiments were performed in triplicate.

#### 3.7.5. Particle Size

The microscopic measurement of the particle size distribution within the powders was conducted using the LEICA Image Processing and Analysis System (Leica Q500MC, Leica Cambridge Ltd., Cambridge, UK). In total, 350 particles per powder sample were investigated and described in terms of their length, width, perimeter, surface area, and roundness. All experiments were performed in three replicates.

#### 3.7.6. Powder Thermal Properties—Differential Scanning Calorimetry

In this analysis, approximately 2–5 mg of each powder was examined in the temperature range of 25 °C to 300 °C. For differential scanning calorimetry (DSC) measurements, the Mettler Toledo DSC 821e thermal analysis system with the STARe thermal analysis program V6.0 (Mettler Inc., Schwerzenbach, Switzerland) was applied. In the analysis, the heating rate was 5 °C min^−1^, while argon was used as a carrier gas at a flow rate of 10 L/h. All experiments were performed in three replicates.

#### 3.7.7. Powder Crystallographic Properties—X-Ray Diffraction

The physical state of the powder was evaluated by X-ray powder diffraction (XRPD). The Bruker D8 advance X-ray powder diffractometer (Bruker AXS GmbH, Karlsruhe, Germany) with Cu K λI radiation (λ = 1.5406 Å) and a VÅNTEC-1 detector (Bruker AXS GmbH) were used for the analysis of the diffraction patterns. The scanning of samples was performed at 40 kV and 40 mA. The angular range was 3–40° 2θ, with an increment time of 0.1 s and increment size of 0.007°. All operations, including Kα2 stripping, background removal, and the smoothing of the area under the diffractogram peak, were performed using the DIFFRACplus EVA software V12.0. All experiments were performed in three replicates.

### 3.8. HPLC Analyses of Liquid Feeds and Corresponding Powders

The concentrations of individual anthocyanins and flavonoids in the liquid feeds and powders were determined using the HPLC-DAD method. Prior to HPLC analysis, the powders were dissolved in methanol at an S/L ratio of 1:10 (*w*/*v*). Extraction was performed on a shaker (GFL 3015, Schuttel Apparat Shakers, Burgwedel, Germany) in the dark, at room temperature, for 24 h. After extraction, the solution was filtrated, and the obtained extracts were placed into a glass bottle and stored until further analysis. The liquid feeds were filtered through a 0.45 μm cellulose filter and transferred to a vial prior to injection. Analyses were carried out on an Agilent series 1200 RR HPLC instrument (Agilent, Waldbronn, Germany), with a DAD detector, on a reverse-phase Lichrospher RP-18 (Agilent) analytical column (250 mm × 4 mm i.d., 5 µm particle size). The mobile phase for anthocyanin analysis consisted of solvent A (10% of formic acid in water) and solvent B (acetonitrile). Samples were separated by gradient elution according to the following scheme: 1% B 0–0.5 min; 1–7% B 0.5–1 min; 7% B 1–4 min; 7–10% B 4–7.5 min; 10–14% B 7.5–11.5 min; 14–25% B 11.5–15.5 min; 25–40% B 15.5–18.5 min; 40–75% B 18.5–22 min; 75% B 22–25 min. The flow was adjusted to 1 mL/min, and the detection wavelengths were set at 290, 350, and 520 nm. Quantification was performed using the calibration curves of anthocyanin standards, including cyanidin-3-O-galactoside, cyanidin-3-O-glucoside, and cyanidin-3-O-arabinoside. For the analysis of individual flavonoids and phenolic acids, the mobile phase consisted of solvent A (1% solution of orthophosphoric acid in water) and mobile phase B (acetonitrile), using gradient elution as follows: 90–80% A 0–5 min, 80% A 5–20 min, 80–40% A 20–30 min, 40–0% A 30–35 min. The detection wavelengths were set at 260 and 350 nm, and the flow rate was 1 mL/min. The amounts of rutin, hyperoside, isoquercitrin, and chlorogenic acid were calculated using calibration curves. The content of all individual phenolic compounds was expressed per mL of methanolic extract. Results are shown as the mean value ± standard deviation (*N* = 3).

### 3.9. Statistical Analysis

The experiments were conducted three times, and the results are presented as the mean value with the standard deviation (±SD). Differences were considered significant when *p* ≤ 0.05. To assess the impacts of individual factors on the observed properties, a one-way ANOVA was performed, followed by Tukey’s HSD post hoc test to identify differences between the mean values (STATISTICA v. 8).

## 4. Conclusions

This study demonstrates that aronia fruit dust, a by-product of the filter tea industry, can be valorized for the production of anthocyanin-containing powders using an integrated ultrasound-assisted extraction and spray-drying approach. The effects of citric, malic, and ascorbic acids, in combination with maltodextrin, were evaluated with respect to selected physical and chemical properties of the obtained powders. The results show that acid addition influenced the process performance and powder characteristics, although the effects were formulation-dependent. Improvements in process efficiency and moisture content were observed in several formulations, but not uniformly across all samples. The addition of organic acids also affected the particle size distribution and bulk density, with citric and malic acids generally yielding powders with smaller and more uniform particles compared to ascorbic acid-containing formulations. Changes in reconstitution-related properties were noted; however, no generalized improvement in solubility could be concluded for any system. Regarding anthocyanins, a decrease in the concentration of the predominant compound (Cya-Gal) was observed after spray drying, although the extent of reduction varied among formulations and could not be described as uniform. No definitive evidence was obtained to confirm the enhanced stabilization of anthocyanins by ascorbic acid under the applied conditions.

This study is subject to several limitations. Mechanistic insights into the role of acidification in anthocyanin stability, particle formation, and matrix interactions remain limited due to the lack of targeted analyses (e.g., detailed thermal characterization, interfacial or rheological measurements). In addition, the absence of storage stability studies prevents conclusions regarding long-term powder behavior. Future work should therefore focus on the systematic investigation of feed properties (e.g., viscosity, pH), controlled drying conditions, and comprehensive stability assessments during storage, alongside the use of advanced analytical techniques to better elucidate the structure–function relationships in these systems.

## Figures and Tables

**Table 1:** Process efficiency.

Powder	P100	P1	P2	P3	P4	P5	P6	P7
Process efficiency (%)	58.9	74.0	70.3	76.4	74.5	78.5	73.2	75.5

**Table 2:** Physical properties of the AFD powders.

Powder	Moisture Content (%)	Hygroscopicity 48 h (%)	Hygroscopicity 120 h (%)	Bulk Density (mg/mL)
P100	5.46 ± 0.05	13.28 ± 0.50	16.13 ± 0.70	90.62 ± 0.04
P1	5.25 ± 0.30	12.28 ± 0.70	14.55 ± 0.70	73.68 ± 0.04
P2	4.69 ± 0.20	13.26 ± 0.70	15.68 ± 0.50	77.50 ± 0.05
P3	3.39 ± 0.10	13.27 ± 0.90	16.08 ± 0.10	55.29 ± 0.06
P4	4.22 ± 0.30	11.96 ± 0.50	14.38 ± 0.50	77.50 ± 0.07
P5	4.81 ± 0.30	12.63 ± 0.50	15.94 ± 0.90	66.22 ± 0.06
P6	4.74 ± 0.20	11.48 ± 0.10	14.58 ± 0.50	56.67 ± 0.02
P7	4.90 ± 0.10	13.56 ± 0.50	16.60 ± 0.50	56.84 ± 0.02

**Table 3:** WSI and WAI of the AFD powders.

Powder	WSI (%)	WAI (g/g)
P100	91.27 ± 0.80	0.82 ± 0.01
P1	82.54 ± 0.60	0.75 ± 0.02
P2	87.24 ± 0.50	0.82 ± 0.01
P3	83.74 ± 0.50	0.78 ± 0.01
P4	86.49 ± 0.60	0.80 ± 0.03
P5	86.81 ± 0.80	0.80 ± 0.02
P6	84.75 ± 0.80	0.78 ± 0.02
P7	83.75 ± 0.80	0.78 ± 0.01

**Table 4:** Size distributions of the AFD powders.

Powder	D 0.1 (µm)	D 0.5 (µm)	D 0.9 (µm)	SSA (m^2^/g)
P100	3.177	8.633	23.205	0.929
P1	2.618	6.666	14.839	1.170
P2	2.604	7.693	18.887	1.120
P3	2.708	7.390	17.471	1.100
P4	2.945	8.103	19.257	1.010
P5	2.517	6.382	14.235	1.220
P6	3.141	9.060	25.834	0.912
P7	3.134	9.126	32.301	0.902

**Table 5:** Concentrations (mg/mL) of anthocyanins in the AFD liquid feeds and powders.

Liquid Feed	Cya-Gal	Cya-Glu	Cya-Ara	Powder	Cya-Gal	Cya-Glu	Cya-Ara
L020	0.62 ± 0.04 ^b^	0.08 ± 0.00 ^b^	0.33 ± 0.04 ^b^	P020	0.19 ± 0.01 ^cd^	0.03 ± 0.00 ^d^	0.10 ± 0.01 ^cd^
L040	0.49 ± 0.07 ^b^	0.07 ± 0.01 ^c^	0.26 ± 0.04 ^b^	P040	0.24 ± 0.02 ^c^	0.03 ± 0.00 ^d^	0.13 ± 0.01 ^c^
L100	0.55 ± 0.03 ^b^	0.07 ± 0.00 ^bc^	0.29 ± 0.03 ^b^	P100	0.20 ± 0.02 ^c^	0.03 ± 0.00 ^d^	0.11 ± 0.01 ^cd^
L1	0.52 ± 0.05 ^b^	0.07 ± 0.00 ^bc^	0.28 ± 0.03 ^b^	P1	0.21 ± 0.02 ^c^	0.03 ± 0.00 ^d^	0.11 ± 0.01 ^cd^
L2	0.49 ± 0.06 ^b^	0.07 ± 0.00 ^c^	0.27 ± 0.03 ^b^	P2	0.19 ± 0.03 ^cd^	0.03 ± 0.00 ^d^	0.10 ± 0.01 ^cd^
L3	0.57 ± 0.04 ^b^	0.08 ± 0.01 ^bc^	0.31 ± 0.02 ^b^	P3	0.21 ± 0.02 ^c^	0.03 ± 0.00 ^d^	0.11 ± 0.02 ^cd^
L4	0.54 ± 0.08 ^b^	0.07 ± 0.01 ^bc^	0.29 ± 0.02 ^b^	P4	0.20 ± 0.03 ^c^	0.03 ± 0.00 ^d^	0.11 ± 0.01 ^cd^
L5	0.50 ± 0.06 ^b^	0.07 ± 0.01 ^c^	0.27 ± 0.02 ^b^	P5	0.18 ± 0.03 ^cd^	0.03 ± 0.00 ^d^	0.10 ± 0.01 ^cd^
L6	0.27 ± 0.02 ^c^	0.04 ± 0.00 ^d^	0.16 ± 0.01 ^c^	P6	0.06 ± 0.01 ^d^	0.01 ± 0.00 ^e^	0.04 ± 0.00 ^d^
L7	0.84 ± 0.10 ^a^	0.12 ± 0.01 ^a^	0.46 ± 0.07 ^a^	P7	0.15 ± 0.01 ^cd^	0.02 ± 0.00 ^de^	0.09 ± 0.01 ^cd^

Different letters indicate statistically significant differences within the same column according to Tukey’s test (*p* < 0.05).

**Table 6:** Concentrations (mg/mL) of phenolic compounds in the AFD liquid feeds and powders.

Liquid Feed	CA	Rutin	Hyperoside	Isoquercitrin	Powder	CA	Rutin	Hyperoside	Isoquercitrin
L020	0.27 ± 0.04 ^b^	0.04 ± 0.00 ^bc^	0.06 ± 0.01 ^c^	0.03 ± 0.00 ^b^	P020	0.06 ± 0.01 ^d^	0.01 ± 0.00 ^cd^	0.01 ± 0.00 ^ef^	0.01 ± 0.00 ^de^
L040	0.22 ± 0.02 ^bc^	0.03 ± 0.00 ^bcd^	0.04 ± 0.00 ^cd^	0.02 ± 0.00 ^bc^	P040	0.07 ± 0.01 ^d^	0.02 ± 0.00 ^cd^	0.01 ± 0.00 ^ef^	0.01 ± 0.00 ^d^
L100	0.23 ± 0.02 ^bc^	0.04 ± 0.00 ^bcd^	0.05 ± 0.00 ^cd^	0.03 ± 0.00 ^b^	P100	0.06 ± 0.01 ^d^	0.01 ± 0.00 ^cd^	0.01 ± 0.00 ^ef^	0.01 ± 0.00 ^de^
L1	0.22 ± 0.03 ^bc^	0.03 ± 0.00 ^bcd^	0.04 ± 0.01 ^cd^	0.02 ± 0.00 ^bc^	P1	0.07 ± 0.01 ^d^	0.01 ± 0.00 ^cd^	0.01 ± 0.00 ^ef^	0.01 ± 0.00 ^de^
L2	0.22 ± 0.03 ^bc^	0.34 ± 0.04 ^a^	0.44 ± 0.02 ^a^	0.03 ± 0.00 ^b^	P2	0.06 ± 0.01 ^d^	0.02 ± 0.00 ^cd^	0.01 ± 0.00 ^ef^	0.01 ± 0.00 ^de^
L3	0.23 ± 0.01 ^bc^	0.33 ± 0.03 ^a^	0.05 ± 0.00 ^cd^	0.03 ± 0.00 ^b^	P3	0.06 ± 0.01 ^d^	0.02 ± 0.00 ^cd^	0.01 ± 0.00 ^ef^	0.01 ± 0.00 ^de^
L4	0.22 ± 0.03 ^bc^	0.03 ± 0.00 ^bcd^	0.04 ± 0.00 ^cd^	0.03 ± 0.00 ^b^	P4	0.05 ± 0.01 ^d^	0.01 ± 0.00 ^cd^	0.01 ± 0.00 ^ef^	0.01 ± 0.00 ^de^
L5	0.22 ± 0.01 ^bc^	0.03 ± 0.00 ^bcd^	0.04 ± 0.00 ^cd^	0.03 ± 0.00 ^b^	P5	0.06 ± 0.00 ^d^	0.02 ± 0.00 ^cd^	0.01 ± 0.00 ^ef^	0.01 ± 0.00 ^de^
L6	0.19 ± 0.01 ^c^	0.01 ± 0.00 ^cd^	0.03 ± 0.00 ^de^	0.02 ± 0.00 ^c^	P6	0.03 ± 0.00 ^d^	0.01 ± 0.00 ^d^	0.00 ± 0.00 ^f^	0.00 ± 0.00 ^e^
L7	0.47 ± 0.05 ^a^	0.06 ± 0.01 ^b^	0.10 ± 0.02 ^b^	0.05 ± 0.01 ^a^	P7	0.06 ± 0.00 ^d^	0.01 ± 0.00 ^cd^	0.01 ± 0.00 ^ef^	0.01 ± 0.00 ^de^

Different letters indicate statistically significant differences within the same column according to Tukey’s test (*p* < 0.05).

**Table 7:** Different formulations of liquid feeds and corresponding powders.

Powder Designation	Liquid Feed Designation	pH Value	Excipients Added			
			MD 19.7 DE	Citric Acid	Malic Acid	Ascorbic Acid
P020	L020	4.38	20%	-	-	-
P040	L040	4.35	40%	-	-	-
P100	L100	4.33	100%	-	-	-
P1	L1	4.18	150%	-	-	-
P2	L2	3.92	150%	4%	-	-
P3	L3	3.76	150%	8%	-	-
P4	L4	3.93	150%	-	4%	-
P5	L5	3.82	150%	-	8%	-
P6	L6	4.13	150%	-	-	4%
P7	L7	4.04	150%	-	-	8%

**Table 8:** Total phenol (TP) content in AFD extracts.

Sample	TP (mg/mL)	TP (mg/g AFD)	TP Mean SD	TP Test
60 min	101.778	44.95978	44.96 ± 6.24	b
80 min	99.7042	61.2599	61.26 ± 4.41	a
100 min	95.271	62.70667	62.71 ± 8.52	a
120 min	102.9218	59.37311	59.37 ± 3.81	ab

Different letters indicate statistically significant differences within the same column according to Tukey’s test (*p* < 0.05).

**Table 9:** Total flavonoid (TF) content in AFD extracts.

Sample	TF (mg/mL)	TF (mg/g AFD)	TF Mean SD	TF Test
60 min	25.4445	8.588936	8.59 ± 0.53	a
80 min	24.92605	9.778216	9.78 ± 0.80	a
100 min	23.81775	10.55304	10.55 ± 0.65	a
120 min	25.73045	9.712479	9.71 ± 1.32	a

The letter a indicates statistically significant differences within the same column according to Tukey’s test (*p* < 0.05).

**Table 10:** Total anthocyanin (Cya-3-glu) content in AFD extracts.

Sample	TA (mg Cya-3-Glu/g AFD)	TA Mean SD	TA Test
60 min	10.06	10.06 ± 1.50	a
80 min	11.72	11.72 ± 0.63	a
100 min	11.84	11.84 ± 0.83	a
120 min	11.48	11.48 ± 1.61	a

The letter a indicates statistically significant differences within the same column according to Tukey’s test (*p* < 0.05).

**Table 11:** HPLC-DAD analysis of dominant anthocyanins in AFD extracts.

Sample	Cya-Gal (mg/g AFD)	Cya-Glu (mg/g AFD)	Cya-Ara (mg/g AFD)
60 min	4.10	0.51	2.10
80 min	4.11	0.48	2.14
100 min	4.74	0.59	2.42
120 min	4.26	0.54	2.17

**Table 12:** HPLC-DAD analysis of dominant phenols in AFD extracts.

Sample	Chlorogenic Acid (mg/g AFD)	Rutin (mg/g AFD)	Hyperoside (mg/g AFD)	Isoquercitrin (mg/g AFD)
60 min	1.86	0.29	0.27	0.15
80 min	1.8	0.29	0.28	0.16
100 min	2.03	0.35	0.33	0.18
120 min	1.93	0.37	0.33	0.19

## Data Availability

Data are contained within the article.

## Abbreviations

The following abbreviations are used in this manuscript:

AFD	Aronia fruit dust
UAE	Ultrasound-assisted extraction
RH	Relative humidity
MD	Maltodextrin
WAI	Water adsorption index
WSI	Water solubility index
SSA	Specific surface area
XRPD	X-ray powder diffraction
DSC	Differential scanning calorimetry
